# Supplementary material for: Discovery of beta-lactamase CMY-10 inhibitors for combination therapy against multi-drug resistant Enterobacteriaceae
Source: PLoS One. 2021 Jan 15;16(1):e0244967. doi: 10.1371/journal.pone.0244967 (PMC7810305; doi:10.1371/journal.pone.0244967)
Supplement: S1 Table — (DOCX) [file pone.0244967.s001.docx]

**S1 Table.** BL-activity and LGFE scores of lead compounds identified as enhancers

| **S. No.** | **Compound** | **BL-activity u/mg** | **LGFE** |
| --- | --- | --- | --- |
| 4 | 17146363 | 0.260954 | -8.44 |
| 5 | 12728806 | 0.28465 | -8.49 |
| 7 | 32025297 | 0.176029 | -9.34 |
| 9 | 49715622 | 0.108729 | -9.41 |
| 10 | 5920960 | 0.160665 | -9.43 |
| 12 | 5553496 | 0.133851 | -8.61 |
| 17 | ‎5146436‎ | 0.100469 | -11.0 |
| 20 | 5648011 | 0.103682 | -11.4 |
| 34 | 6882557 | 0.150752 | -9.70 |
| 69 | 7604879 | 0.086792 | -9.57 |
| 71 | 7458478 | 0.137233 | -9.58 |
