# Supplementary material for: Discovery of beta-lactamase CMY-10 inhibitors for combination therapy against multi-drug resistant Enterobacteriaceae
Source: PLoS One. 2021 Jan 15;16(1):e0244967. doi: 10.1371/journal.pone.0244967 (PMC7810305; doi:10.1371/journal.pone.0244967)
Supplement: S2 Table — (DOCX) [file pone.0244967.s002.docx]

**S2 Table.** Epsiolmeter test results for commercially available beta-lactam antibiotics against MDR clinical isolates.

| **Bacterial strains** | **AML** | | **CFM** | | **CS** | | **CTX** | | **FEP** | | **ATM** | | **ETP** | | **IP** | | **MP** | |
| --- | --- | --- | --- | --- | --- | --- | --- | --- | --- | --- | --- | --- | --- | --- | --- | --- | --- | --- |
|  | ***MIC*** *µg/ml* | **Sensit-ivity** | ***MIC*** *µg/ml* | **Sensit-ivity** | ***MIC*** *µg/ml* | **Sensit-ivity** | ***MIC*** *µg/ml* | **Sensit-ivity** | ***MIC*** *µg/ml* | **Sensit-ivity** | ***MIC*** *µg/ml* | **Sensit-ivity** | ***MIC*** *µg/ml* | **Sensit-ivity** | ***MIC*** *µg/ml* | **Sensit-ivity** | ***MIC*** *µg/ml* | **Sensit-ivity** |
| *E. cloacae* | 256 | R | 256 | S | 256 | S | 16 | R | 16 | R | 256 | S | 32 | R | 32 | S | 32 | S |
|  | 190 | R | 190 | S | 190 | S | 12 | R | 12 | R | 190 | S | 24 | R | 24 | R | 24 | S |
|  | 128 | R | 128 | S | 128 | S | 8 | R | 8 | R | 128 | S | 16 | R | 16 | R | 16 | R |
|  | 96 | R | 96 | S | 96 | R | 6 | R | 6 | R | 96 | S | 12 | R | 12 | R | 12 | R |
|  | 64 | R | 64 | R | 64 | R | 4 | R | 4 | R | 64 | R | 8 | R | 8 | R | 8 | R |
|  | 48 | R | 48 | R | 48 | R | 3 | R | 3 | R | 48 | R | 6 | R | 6 | R | 6 | R |
|  | 32 | R | 32 | R | 32 | R | 2 | R | 2 | R | 32 | R | 4 | R | 4 | R | 4 | R |
|  | 24 | R | 24 | R | 24 | R | 1.5 | R | 1.5 | R | 24 | R | 3 | R | 3 | R | 3 | R |
|  | 16 | R | 16 | R | 16 | R | 1 | R | 1 | R | 16 | R | 2 | R | 2 | R | 2 | R |
|  | 12 | R | 12 | R | 12 | R | 0.75 | R | 0.75 | R | 12 | R | 1 | R | 1 | R | 1 | R |
|  | 6 | R | 6 | R | 6 | R | 0.5 | R | 0.5 | R | 6 | R | 0.75 | R | 0.75 | R | 0.75 | R |
|  | 4 | R | 4 | R | 4 | R | 0.38 | R | 0.38 | R | 4 | R | 0.5 | R | 0.5 | R | 0.5 | R |
|  | 3 | R | 3 | R | 3 | R | 0.25 | R | 0.25 | R | 3 | R | 0.38 | R | 0.38 | R | 0.38 | R |
|  | 2 | R | 2 | R | 2 | R |  |  |  |  | 2 | R | 0.25 | R | 0.25 | R | 0.25 | R |
|  | 1.5 | R | 1.5 | R | 1.5 | R |  |  |  |  | 1.5 | R | 0.19 | R | 0.19 | R | 0.19 | R |
|  | 1 | R | 1 | R | 1 | R |  |  |  |  | 1 | R | 0.5 | R | 0.5 | R | 0.5 | R |
|  | 0.75 | R | 0.75 | R | 0.75 | R |  |  |  |  | 0.75 | R | 0.1/.002 | R | 0.1/0.002 | R | 0.1/0.002 | R |
|  | 0.5 | R | 0.5 | R | 0.5 | R |  |  |  |  | 0.5 | R |  |  |  |  |  |  |
|  | 0.19 | R | 0.19 | R | 0.19 | R |  |  |  |  | 0.19 | R |  |  |  |  |  |  |
|  | 0.5 | R | 0.5 | R | 0.5 | R |  |  |  |  | 0.5 | R |  |  |  |  |  |  |
|  | 0.1/ 0.016 | R | 0.1/0.016 | R | 0.1/ 0.016 | R |  |  |  |  | 0.1/.016 | R |  |  |  |  |  |  |
| ***E. agglumerans*** | **256** | **S** | **256** | **S** | **256** | **S** | **16** | **R** | **16** | **S** | **256** | **S** | **32** | **R** | **32** | **R** | **32** | **R** |
|  | 190 | S | 190 | S | 190 | S | 12 | R | 12 | S | 190 | S | 24 | R | 24 | S | 24 | S |
|  | 128 | S | 128 | S | 128 | S | 8 | R | 8 | S | 128 | S | 16 | R | 16 | S | 16 | S |
|  | 96 | S | 96 | S | 96 | S | 6 | R | 6 | S | 96 | S | 12 | R | 12 | R | 12 | R |
|  | 64 | R | 64 | S | 64 | R | 4 | R | 4 | S | 64 | R | 8 | R | 8 | R | 8 | R |
|  | 48 | R | 48 | R | 48 | R | 3 | R | 3 | S | 48 | R | 6 | R | 6 | R | 6 | R |
|  | 32 | R | 32 | R | 32 | R | 2 | R | 2 | S | 32 | R | 4 | R | 4 | R | 4 | R |
|  | 24 | R | 24 | R | 24 | R | 1.5 | R | 1.5 | S | 24 | R | 3 | R | 3 | R | 3 | R |
|  | 16 | R | 16 | R | 16 | R | 1 | R | 1 | S | 16 | R | 2 | R | 2 | R | 2 | R |
|  | 12 | R | 12 | R | 12 | R | 0.75 | R | 0.75 | S | 12 | R | 1 | R | 1 | R | 1 | R |
|  | 6 | R | 6 | R | 6 | R | 0.5 | R | 0.5 | S | 6 | R | 0.75 | R | 0.75 | R | 0.75 | R |
|  | 4 | R | 4 | R | 4 | R | 0.38 | R | 0.38 | S | 4 | R | 0.5 | R | 0.5 | R | 0.5 | R |
|  | 3 | R | 3 | R | 3 | R | 0.25 | R | 0.25 | S | 3 | R | 0.38 | R | 0.38 | R | 0.38 | R |
|  | 2 | R | 2 | R | 2 | R |  |  |  |  | 2 | R | 0.25 | R | 0.25 | R | 0.25 | R |
|  | 1.5 | R | 1.5 | R | 1.5 | R |  |  |  |  | 1.5 | R | 0.19 | R | 0.19 | R | 0.19 | R |
|  | 1 | R | 1 | R | 1 | R |  |  |  |  | 1 | R | 0.5 | R | 0.5 | R | 0.5 | R |
|  | 0.75 | R | 0.75 | R | 0.75 | R |  |  |  |  | 0.75 | R | 0.1/.002 | R | 0.1/.002 | R | 0.1/0.002 | R |
|  | 0.5 | R | 0.5 | R | 0.5 | R |  |  |  |  | 0.5 | R | 0.5 | R |  |  |  |  |
|  | 0.19 | R | 0.19 | R | 0.19 | R |  |  |  |  | 0.19 | R | 0.19 | R |  |  |  |  |
|  | 0.5 | R | 0.5 | R | 0.5 | R |  |  |  |  | 0.5 | R | 0.5 | R |  |  |  |  |
|  | 0.1/ 0.016 | R | 0.1/ 0.016 | R | 0.1/ 0.016 | R |  |  |  |  | 0.1/.016 | R | 0.1 | R |  |  |  |  |
| ***E. alvei*** | **256** | **S** | **256** | **S** | **256** | **S** | **16** | **R** | **16** | **R** | **256** | **S** | **32** | **R** | **32** | **S** | **32** | **S** |
|  | 190 | S | 190 | S | 190 | S | 12 | R | 12 | R | 190 | S | 24 | R | 24 | S | 24 | S |
|  | 128 | S | 128 | S | 128 | S | 8 | R | 8 | R | 128 | S | 16 | R | 16 | S | 16 | S |
|  | 96 | S | 96 | S | 96 | S | 6 | R | 6 | R | 96 | S | 12 | R | 12 | S | 12 | R |
|  | 64 | S | 64 | S | 64 | R | 4 | R | 4 | R | 64 | S | 8 | R | 8 | R | 8 | R |
|  | 48 | R | 48 | S | 48 | R | 3 | R | 3 | R | 48 | R | 6 | R | 6 | R | 6 | R |
|  | 32 | R | 32 | R | 32 | R | 2 | R | 2 | R | 32 | R | 4 | R | 4 | R | 4 | R |
|  | 24 | R | 24 | R | 24 | R | 1.5 | R | 1.5 | R | 24 | R | 3 | R | 3 | R | 3 | R |
|  | 16 | R | 16 | R | 16 | R | 1 | R | 1 | R | 16 | R | 2 | R | 2 | R | 2 | R |
|  | 12 | R | 12 | R | 12 | R | 0.75 | R | 0.75 | R | 12 | R | 1 | R | 1 | R | 1 | R |
|  | 6 | R | 6 | R | 6 | R | 0.5 | R | 0.5 | R | 6 | R | 0.75 | R | 0.75 | R | 0.75 | R |
|  | 4 | R | 4 | R | 4 | R | 0.38 | R | 0.38 | R | 4 | R | 0.5 | R | 0.5 | R | 0.5 | R |
|  | 3 | R | 3 | R | 3 | R | 0.25 | R | 0.25 | R | 3 | R | 0.38 | R | 0.38 | R | 0.38 | R |
|  | 2 | R | 2 | R | 2 | R |  |  |  |  | 2 | R | 0.25 | R | 0.25 | R | 0.25 | R |
|  | 1.5 | R | 1.5 | R | 1.5 | R |  |  |  |  | 1.5 | R | 0.19 | R | 0.19 | R | 0.19 | R |
|  | 1 | R | 1 | R | 1 | R |  |  |  |  | 1 | R | 0.5 | R | 0.5 | R | 0.5 | R |
|  | 0.75 | R | 0.75 | R | 0.75 | R |  |  |  |  | 0.75 | R | 0.1/.002 | R | 0.1/.002 | R | 0.1/0.002 | R |
|  | 0.5 | R | 0.5 | R | 0.5 | R |  |  |  |  | 0.5 | R |  |  |  |  |  |  |
|  | 0.19 | R | 0.19 | R | 0.19 | R |  |  |  |  | 0.19 | R |  |  |  |  |  |  |
|  | 0.5 | R | 0.5 | R | 0.5 | R |  |  |  |  | 0.5 | R |  |  |  |  |  |  |
|  | 0.1 | R | 0.1/ 0.016 | R | 0.1/ 0.016 | R |  |  |  |  | 0.1/.016 | R |  |  |  |  |  |  |

**List of antibiotics and codes:** Penicillin (AML), Cefixime (CFM), Ceftriaxone (CF), Cefotaxime (CTX), Cefepime (FEP), Azithromycin (ATM), Ertapenem (ETP), Imipenem (IP), Meropenem (MP)

S= SENSITIVE = ≥17 mm

I=INTERMEDIATE = 15 mm – 17 mm

R= RESISTANCE = ≤ 14 mm
