# Supplementary material for: Discovery of beta-lactamase CMY-10 inhibitors for combination therapy against multi-drug resistant Enterobacteriaceae
Source: PLoS One. 2021 Jan 15;16(1):e0244967. doi: 10.1371/journal.pone.0244967 (PMC7810305; doi:10.1371/journal.pone.0244967)
Supplement: S3 Table — (DOCX) [file pone.0244967.s003.docx]

**S3 Table.** Antibacterial activity of lead compounds against ATCC bacterial isolates with the zone of inhibition (mm).

| **S. No** | **Chembridge ID** | **BACTERIAL ISOLATES (ATCC)** | | | |  |
| --- | --- | --- | --- | --- | --- | --- |
|  |  | ***Escherichia coli (*ATCC 10536*)*** | ***Enterobacter cloacae (*ATCC 13047)** | ***Enterobacter* *agglomerans* (ATCC 31901)** | ***Enterobacter alvei* (ATCC 51815)** |  |
|  |  | **Zone of inhibition (mm)** | | | |  |
|  |  | **M ± SD** | **M ± SD** | **M ± SD** | **M ± SD** |  |
| **1** | **6096429** | 0 ± 0 | 8.3 ±0.4 | 13.3 ±0.4 | 8.3 ±0.4 |  |
| **5** | **12728806** | 11.6 ±0.4 | 13.3 ±0.4 | 8.3 ±0.4 | 4.3 ±0.4 |  |
| **11** | **5524250** | 18.3 ±0.4 | 18.3 ±0.4 | 18.3 ±0.4 | 11.6 ±0.4 |  |
| **26** | **5241230** | 0 ± 0 | 18.3 ±0.4 | 11.6 ±0.4 | 13.3 ±0.4 |  |
| **36** | **77764831** | 4.3 ±0.4 | 0 ± 0 | 4.3 ±0.4 | 0 ± 0 |  |
| **37** | **7989492** | 8.3 ±0.4 | 0 ± 0 | 18.3 ±0.4 | 11.6 ±0.4 |  |
| **47** | **7878453** | 0 ± 0 | 18.3 ±0.4 | 4.3 ±0.4 | 8.3 ±0.4 |  |
| **54** | **7960496** | 0 ± 0 | 8.3 ±0.4 | 8.3 ±0.4 | 11.6 ±0.4 |  |
| **Control** | **Cefixime** | 19.6±0.4 | 19.3 ±0.4 | 19.3 ±0.4 | 19.3 ±0.4 |  |

* M ± SD, Mean ± Standard Deviation, mm, millimeter,
