# Supplementary material for: Discovery of beta-lactamase CMY-10 inhibitors for combination therapy against multi-drug resistant Enterobacteriaceae
Source: PLoS One. 2021 Jan 15;16(1):e0244967. doi: 10.1371/journal.pone.0244967 (PMC7810305; doi:10.1371/journal.pone.0244967)
Supplement: S4 Table — (DOCX) [file pone.0244967.s004.docx]

**S4 Table.** Two dimensional chemical structures of the similarity searched compounds including active compound 11.

| **Compound No.** | **Chemical Structure** |
| --- | --- |
| 1 |  |
| 2 |  |
| 3 |  |
| 4 |  |
| 5 |  |
| 6 |  |
| 7 |  |
| 8 |  |
| 9 |  |
| 10 |  |
| 11 |  |
| 12 |  |
| 13 |  |
| 14 |  |
| 15 |  |
| 16 |  |
| 17 |  |
| 18 |  |
| 19 |  |
| 20 |  |
| 21 |  |
| 22 |  |
| 23 |  |
| 24 |  |
| 25 |  |
| 26 |  |
| 27 |  |
| 28 |  |
| 29 |  |
