# Supplementary material for: Discovery of beta-lactamase CMY-10 inhibitors for combination therapy against multi-drug resistant Enterobacteriaceae
Source: PLoS One. 2021 Jan 15;16(1):e0244967. doi: 10.1371/journal.pone.0244967 (PMC7810305; doi:10.1371/journal.pone.0244967)
Supplement: S5 Table — (DOCX) [file pone.0244967.s005.docx]

**S5 Table.** BL-activity of further screened compounds.

| **Compound No** | **BL actvity unit/ug** |
| --- | --- |
| 1 | 0.14 |
| 2 | 0.16 |
| 3 | 0.16 |
| 4 | 0.15 |
| 5 | 0.11 |
| 6 | 0.14 |
| 7 | 0.14 |
| 8 | 0.13 |
| 9 | 0.37 |
| 10 | 0.11 |
| **11** | 0.07 |
| 12 | 0.17 |
| 13 | 0.15 |
| 14 | 0.10 |
| 15 | 0.45 |
| 16 | 0.17 |
| 17 | 0.11 |
| 18 | 0.19 |
| 19 | 0.74 |
| 20 | 0.06 |
| 21 | 0.26 |
| 22 | 0.09 |
| 23 | 0.27 |
| 24 | 0.13 |
| 25 | 0.11 |
| 26 | 1.5 |
| 27 | 0.66 |
| 28 | 1.1 |
| 29 | 0.14 |
| Control: Cefixime | 0.58 |
