# Supplementary material for: Discovery of beta-lactamase CMY-10 inhibitors for combination therapy against multi-drug resistant Enterobacteriaceae
Source: PLoS One. 2021 Jan 15;16(1):e0244967. doi: 10.1371/journal.pone.0244967 (PMC7810305; doi:10.1371/journal.pone.0244967)
Supplement: S6 Table — (DOCX) [file pone.0244967.s006.docx]

| **S. No** | **BACTERIAL ISOLATES (ATCC) compounds concentration ( 5mg/ml) 50micro gram per disc** | | | |
| --- | --- | --- | --- | --- |
|  | ***Escherichia coli (*ATCC 10536*)*** | ***Enterobacter cloacae (*ATCC 13047)** | ***Enterobacter* *agglomerans* (ATCC 31901)** | ***Enterobacter alvei* (ATCC 51815)** |
|  | **Zone of inhibition (mm)** | | | |
|  | **M ± SD** | **M ± SD** | **M ± SD** | **M ± SD** |
| **1** | 4.6 ±0.4 | 4.3 ±0.4 | 6.6 ± 0.4 | 6.6 ± 0.4 |
| **2** | 4.6 ±0.4 | 4.6 ±0.4 | 4.6 ±0.4 | 8.3 ±0.4 |
| **3** | 5.6 ± 0.4 | 6.6 ± 0.4 | 6.6 ± 0.4 | 6.6 ± 0.4 |
| **4** | 6.6 ± 0.4 | 4.6 ±0.4 | 6.6 ± 0.4 | 8.6 ±0.4 |
| **5** | 8.3 ±0.4 | 6.6 ± 0.4 | 8.3 ±0.4 | 0 ± 0 |
| **6** | 6.6 ± 0.4 | 8.3 ±0.4 | 6.6 ± 0.4 | 4.6 ±0.4 |
| **7** | 8.3 ±0.4 | 6.6 ± 0.4 | 8.6 ±0.4 | 6.6 ± 0.4 |
| **8** | 5.6 ± 0.4 | 4.6 ±0.4 | 0 ± 0 | 5.6 ± 0.4 |
| **9** | 5.6 ± 0.4 | 6.6 ± 0.4 | 4.6 ±0.4 | 6.6 ± 0.4 |
| **10** | 5.6 ± 0.4 | 6.6 ± 0.4 | 6.6 ± 0.4 | 8.3 ±0.4 |
| **11** | 18.3 ±0.4 | 18.3 ±0.4 | 18.3 ±0.4 | 11.6 ±0.4 |
| **12** | 6.6 ± 0.4 | 5.6 ± 0.4 | 4.6 ±0.4 | 4.6 ±0.4 |
| **13** | 5.6 ± 0.4 | 4.6 ±0.4 | 4.6 ±0.4 | 4.6 ±0.4 |
| **14** | 8.6 ±0.4 | 8.3 ±0.4 | 4.6 ±0.4 | 5.6 ± 0.4 |
| **15** | 0 ± 0 | 0 ± 0 | 5.6 ± 0.4 | 4.6 ±0.4 |
| **16** | 4.6 ±0.4 | 4.6 ±0.4 | 4.6 ±0.4 | 8.6 ±0.4 |
| **17** | 6.6 ± 0.4 | 5.6 ± 0.4 | 4.6 ±0.4 | 0 ± 0 |
| **18** | 5.6 ± 0.4 | 6.6 ± 0.4 | 5.6 ± 0.4 | 4.6 ±0.4 |
| **19** | 4.6 ±0.4 | 4.6 ±0.4 | 5.6 ± 0.4 | 6.6 ± 0.4 |
| **20** | 13.3 ±0.4 | 11.6 ±0.4 | 18.3 ±0.4 | 8.3 ±0.4 |
| **21** | 5.6 ± 0.4 | 4.6 ±0.4 | 8.6 ±0.4 | 6.6 ± 0.4 |
| **22** | 8.6 ±0.4 | 8.3 ±0.4 | 4.6 ±0.4 | 8.6 ±0.4 |
| **23** | 4.6 ±0.4 | 4.6 ±0.4 | 8.6 ±0.4 | 4.6 ±0.4 |
| **24** | 8.6 ±0.4 | 8.3 ±0.4 | 4.6 ±0.4 | 8.6 ±0.4 |
| **25** | 4.6 ±0.4 | 4.6 ±0.4 | 4.6 ±0.4 | 4.6 ±0.4 |
| **26** | 0 ± 0 | 0 ± 0 | 4.6 ±0.4 | 4.6 ±0.4 |
| **27** | 4.6 ±0.4 | 4.6 ±0.4 | 5.6 ± 0.4 | 5.6 ± 0.4 |
| **28** | 4.6 ±0.4 | 4.6 ±0.4 | 4.6 ±0.4 | 6.6 ± 0.4 |
| **29** | 5.6 ± 0.4 | 6.6 ± 0.4 | 4.6 ±0.4 | 8.3 ±0.4 |
| **Control** | 19.6±0.4 | 19.3 ±0.4 | 19.3 ±0.4 | 19.3 ±0.4 |

**S6 Table.** Antibacterial activity of similarity searched compounds against ATCC bacterial isolates with the zone of inhibition (mm).

* M ± SD, Mean ± Standard Deviation, mm, millimeter
