# Supplementary figures and images for: Discovery of beta-lactamase CMY-10 inhibitors for combination therapy against multi-drug resistant Enterobacteriaceae
Source: PLoS One. 2021 Jan 15;16(1):e0244967. doi: 10.1371/journal.pone.0244967 (PMC7810305; doi:10.1371/journal.pone.0244967)

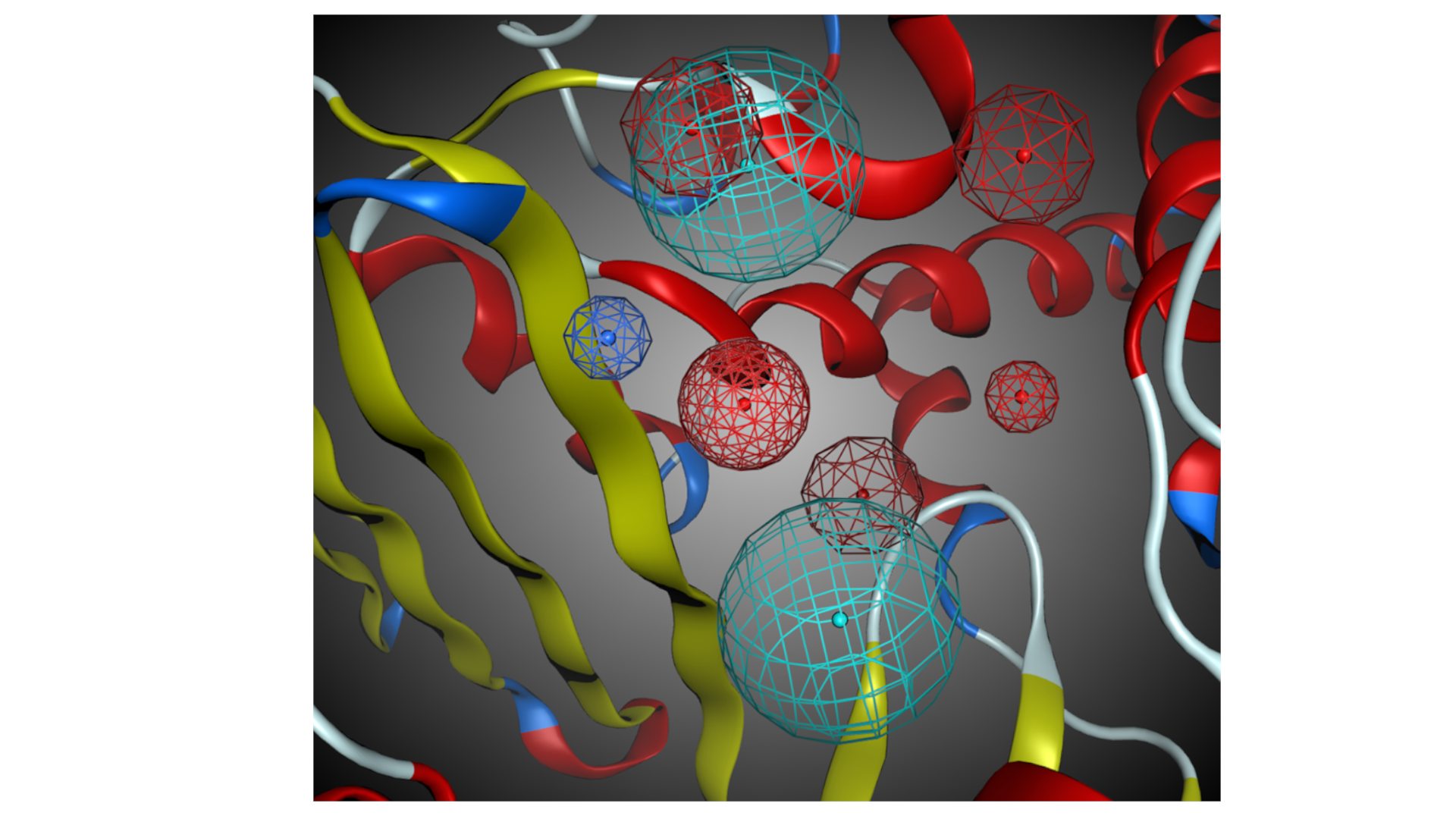

Supplement: S1 Fig — Hydrophobic, hydrogen bond acceptor, hydrogen bond donor and negatively charged features are colored in cyan, red, blue, and dark red, respectively. (TIF) [file pone.0244967.s008.tif]

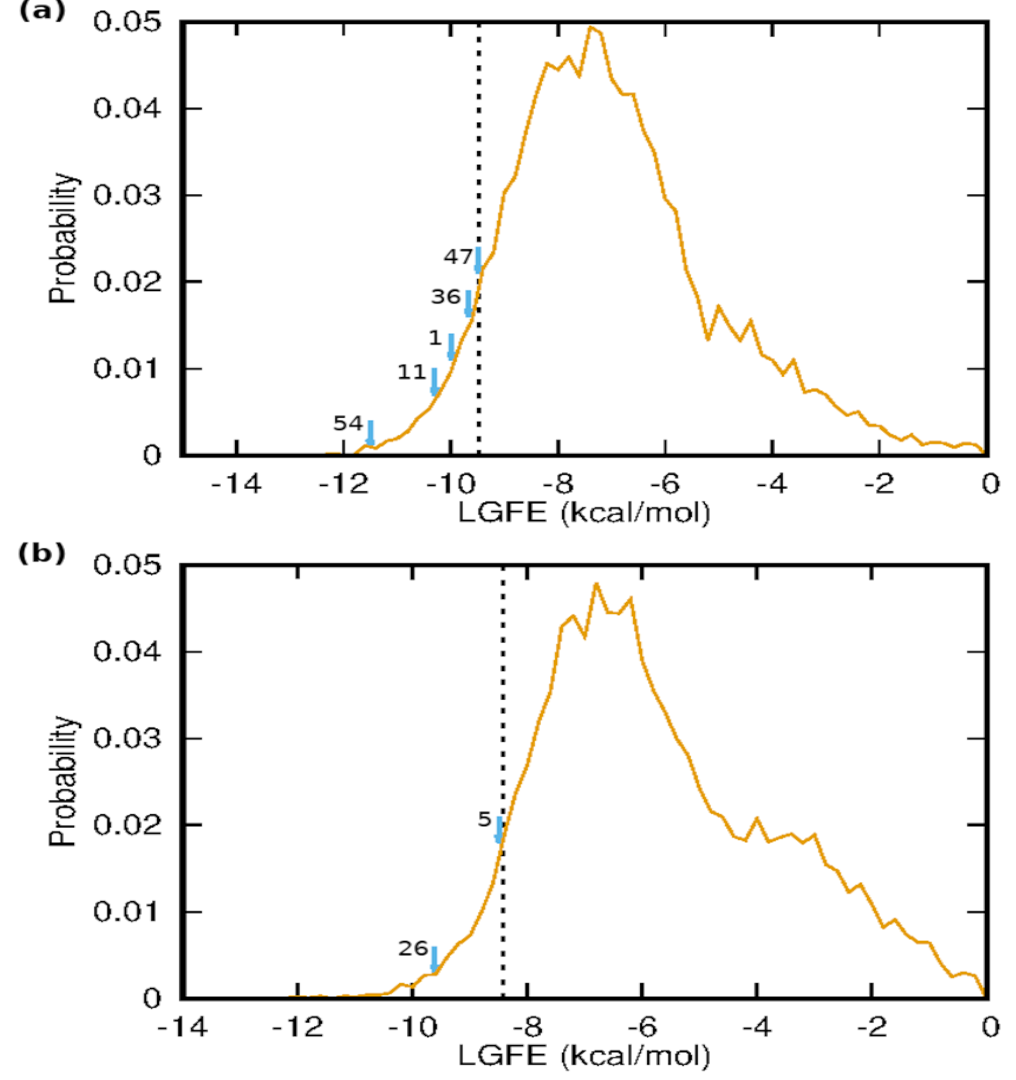

Supplement: S2 Fig — LGFE distributions for top 10,000 ranked compounds from VS for both R2 model (a) and R1-R2 (b) model. The vertical dashed line indicates the LGFE cutoff for selecting the top 500 ranked compounds. The LGFE values for the seven compounds identified as inhibitors are indicated by blue arrows with their compound ID labeled. 5 hits are from the R2 model VS and 2 hits are from the R1-R2 model VS. (TIF) [file pone.0244967.s009.tif]

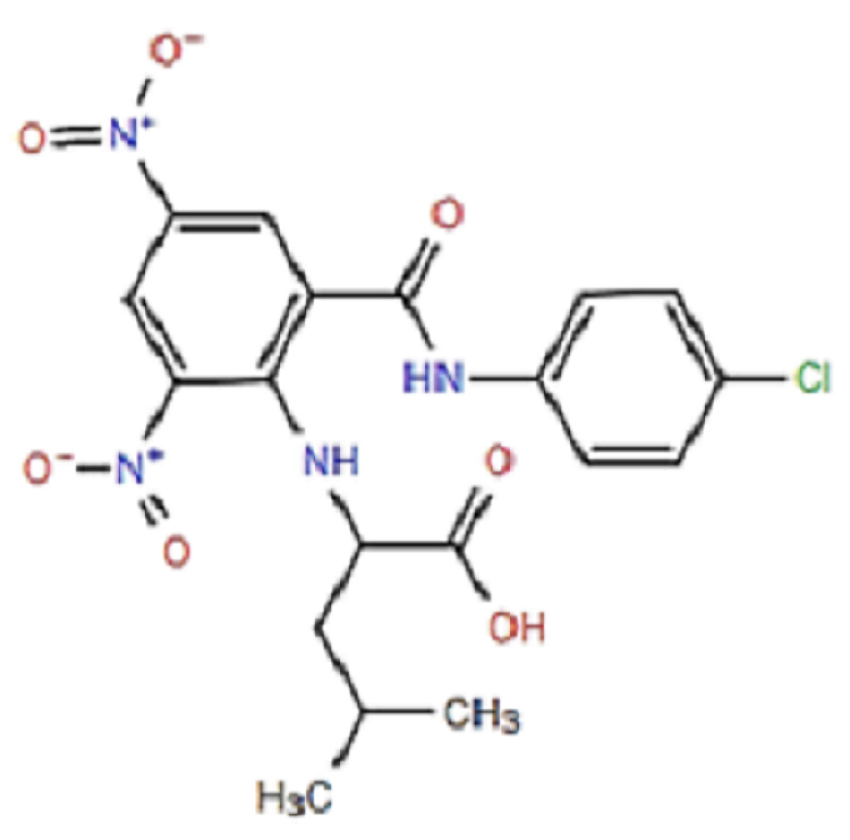

Supplement: S3 Fig — (TIF) [file pone.0244967.s010.tif]

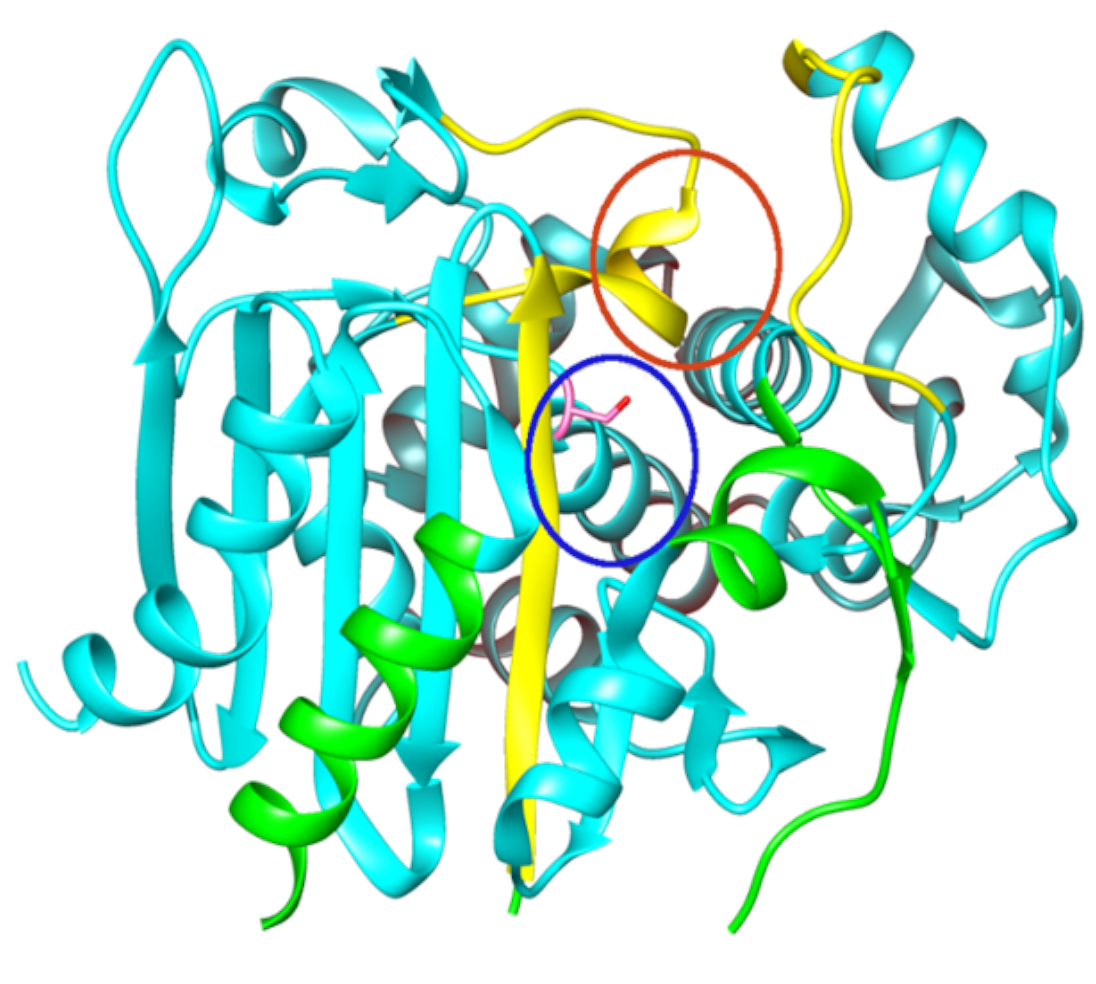

Supplement: S4 Fig — (A) The 3D structure of CMY-10 in ribbon representation. The residues of R1 site (Ω-loop, Gln121 loop and β11) and R2 site (Tyr151 loop, α10 and α11) are in yellow and green, respectively. The binding cavity of R1 site is represented by red ellipse and R2 site is represented by blue ellipse. The nucleophile Ser65 is shown in magenta using ball and stick representation within the blue ellipse. (TIF) [file pone.0244967.s011.tif]

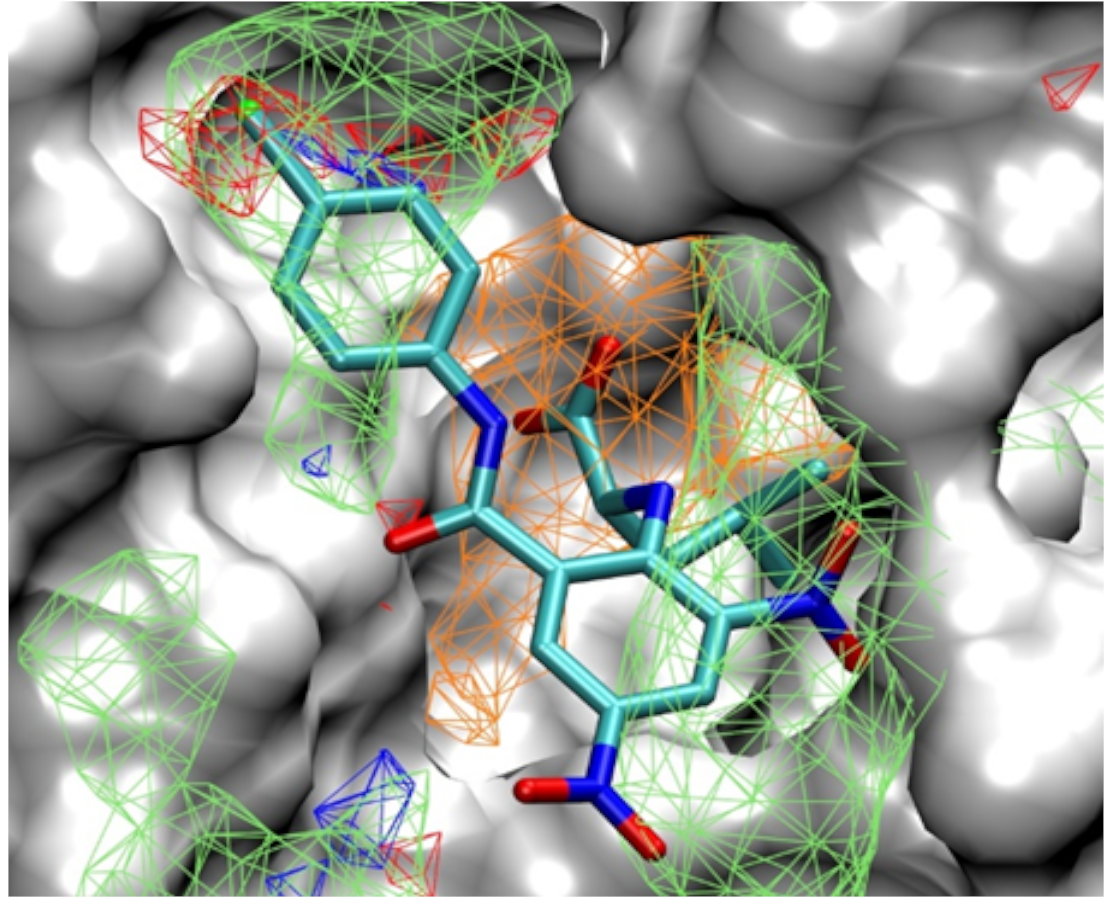

Supplement: S5 Fig — The binding site is shown in the same orientation as in the Fig 2. FragMaps are shown at GFE cutoff -1.0 kcal/mol for apolar (green), hydrogen bonding donor (blue) and acceptor (red) maps and at -1.5 kcal/mol for negatively (orange) charged map. (TIF) [file pone.0244967.s012.tif]
