# Supplementary material for: Discovery of beta-lactamase CMY-10 inhibitors for combination therapy against multi-drug resistant Enterobacteriaceae
Source: PLoS One. 2021 Jan 15;16(1):e0244967. doi: 10.1371/journal.pone.0244967 (PMC7810305; doi:10.1371/journal.pone.0244967)
Supplement: Attachment — Submitted filename: PONE-D-20-14041.pdf [file pone.0244967.s013.pdf]

This is an interesting paper highlighting an innovative approach at antimicrobial resistance of Enterobacteriaceae spp.

The paper is well written and detailed.

However, I have some questions about article:

Authors should explain a potential action mechanism of compound 11

Can author clarify the correlation between lab tests and potential clinical applications?
